# Inappropriate Influence by Industry on *EHP* News Article

**DOI:** 10.1289/ehp.113-1277886

**Published:** 2005-02

**Authors:** Jennifer Sass, Gina Solomon

**Affiliations:** Natural Resources Defense Council Washington, DC, E-mail: jsass@nrdc.org; Natural Resources Defense Council San Francisco, CA

We are disturbed at the recent revelation that, before its publication, an *EHP* Science Selection article on the toxic rocket fuel additive perchlorate (Renner 2002) was substantially revised by a paid consultant to the perchlorate industry to downplay adverse health effects (Danelski 2004). *EHP* contracted freelance journalist Rebecca Renner to write the Science Selection piece to accompany publication of a research article by Greer et al. (2002). The Greer study was partially funded by the Perchlorate Study Group (PSG), a self-described alliance of perchlorate users and manufacturers, including the military contractors Boeing, Lockheed Martin, Aerojet, and Kerr-McGee. It seems apparent that the PSG had a significant financial interest in continued perchlorate use. The study included two authors affiliated with Intertox, Inc., a consulting firm contracted by the PSG. Greer et al. (2002) reported on the effects of perchlorate ingestion by 37 healthy adults over a 2-week period, concluding that

Assuming that the drinking water supply is the only significant source of exposure …, a perchlorate concentration of 180–220 ppb (and possibly much higher) should be of no health concern in iodine-sufficient populations.

The original article by Renner reported that the Greer study results disagreed with a draft toxicologic review released by the U.S. Environmental Protection Agency (EPA) just a few months earlier, recommending no more than 1 ppb perchlorate in drinking water, on the basis of reduced thyroid hormone levels, abnormal brain morphology, and thyroid cancers in young rat pups exposed *in utero* and perinatally to perchlorate (U.S. EPA 2002).

Prior to publication, the *EHP* editors sent Renner’s article to one of the authors of the Greer study, Gay Goodman, of Intertox, Inc., to check the characterization of her study for accuracy. According to an invoice from Intertox to PSG (Pleus 2002), Goodman was paid by the industry for her time spent “editing the *EHP* news article (Renner 2002).” Intertox explained in the invoice that Renner’s article, as originally written, “was potentially very damaging to PSG” (Pleus 2002). The invoice described Intertox’s work: “Dr. Goodman gained the trust of the editor and, through a cooperative process entailing five or more drafts, provided substantial and critical improvements to the article” (Pleus 2002). Renner was never provided an opportunity to review the revised version, yet practically every sentence of her article had been altered (Danelski 2004). In particular, all references to the inconsistency between the drinking water “safe” level recommended by the Greer study and that of the U.S. EPA were scrubbed from the published version, as was the disclosure of the major funding source of the Greer study.

Although the Intertox rewrite of Renner’s news story was titled “Reprieve for Perchlorate: Effects Not a Significant Concern” and the revised story was written to imply that the Greer study proved perchlorate to be less toxic than previously believed, these conclusions are unfounded and misleading. More recent reviews of the Greer study by state agencies, the U.S. EPA, and independent scientists have noted that the low-dose group in the study was too small to have the statistical power to detect a relevant effect (Massachusetts Department of Environmental Protection 2004). Furthermore, the results could not be extrapolated to longer-term exposures, iodine-deficient populations (including 15% of women in the United States), or to the fetus and infant (Hollowell et al. 1998).

The Department of Defense and its military contractors have waged a coordinated assault on the U.S. EPA regulatory recommendation of no more than 1 ppb perchlorate in drinking water (Beeman and Danelski 2004; Sass 2004). It now appears that *EHP* unwittingly became part of that campaign to manipulate the public perception of perchlorate and avoid strict regulatory standards. In other efforts, the Department of Defense and PSG pushed for the National Academies to provide a review of the U.S. EPA recommendations (Renner 2003), with the report expected in late January 2005. A paid consultant to Lockheed Martin was initially appointed to the Academies’ scientific committee, but was later asked to resign when previously undisclosed evidence of financial conflicts emerged (Waldman 2004).

We applaud *EHP*’s excellent reputation for scientific quality and integrity. However, in this case we believe the evidence shows that *EHP* was unwittingly used as part of a deliberate campaign to undermine a health policy position taken by a sister agency. We request that the *EHP* editors implement policies to ensure that any alterations made to a manuscript be clearly marked and reviewed by the author before going to press. *EHP* has indicated that it may add another layer of editing by outside scientists to the Science Selection editing process in response to this situation (Beeman and Danelski 2004). We strongly discourage the use of outside reviewers for news stories and suggest instead that *EHP* rely on the expertise of its editorial staff and news reporters, with limited use of outside reviewers for accuracy checks only. We applaud the recent *EHP* policy to ban from publishing for 3 years any author who fails to disclose financial conflicts (EHP 2004). If *EHP* applied such a requirement to news articles, it would bring needed transparency to efforts like those of Intertox consultant Gay Goodman, who effectively ghost authored the Renner article (with permission of *EHP* editors) without disclosure to the *EHP* readership.
